# CAN*L*ead: Canada as a Phase 1 Clinical Trial Leader — Opportunities, Initiatives, and Collaborative Innovation to Elevate National Competitiveness

**DOI:** 10.1093/oncolo/oyag190

**Published:** 2026-05-22

**Authors:** Ashley A Adile, Gagan Sran, Philippe L Bedard, Lindsay Carlsson, Jeffrey Doi, Nancy Drummond-Ivars, Sevan Hakgor, Amy Henderson, John F Hilton, Philip David Josephy, Jim Kremidas, Jacqueline Limoges, Daniel J Renouf, Fiona Ross, Abdulazeez Salawu, Ramy Saleh, Lillian L Siu, Isabelle Voccia, Hanusya G Lewis, Miney Paquette, Albiruni R Abdul Razak, Anna Spreafico

**Affiliations:** Drug Development Program (Phase I), Princess Margaret Cancer Centre, University Health Network,Toronto, ON M5G 2M9, Canada; Drug Development Program (Phase I), Princess Margaret Cancer Centre, University Health Network,Toronto, ON M5G 2M9, Canada; Drug Development Program (Phase I), Princess Margaret Cancer Centre, University Health Network,Toronto, ON M5G 2M9, Canada; Drug Development Program (Phase I), Princess Margaret Cancer Centre, University Health Network,Toronto, ON M5G 2M9, Canada; Drug Development Program (Phase I), Princess Margaret Cancer Centre, University Health Network,Toronto, ON M5G 2M9, Canada; Oncology Clinical Trials (Solid Tumor) Program, The Ottawa Hospital Research Institute, Ottawa, ON K1H 8L6, Canada; Drug Development Program (Phase I), Princess Margaret Cancer Centre, University Health Network,Toronto, ON M5G 2M9, Canada; Oncology Clinical Trials (Solid Tumor) Program, The Ottawa Hospital Research Institute, Ottawa, ON K1H 8L6, Canada; Oncology Clinical Trials (Solid Tumor) Program, The Ottawa Hospital Research Institute, Ottawa, ON K1H 8L6, Canada; GIST Sarcoma Life Raft Group Canada, Guelph, ON N1G 2G2, Canada; Association for MultiSite Research Corporations, Lake Mary, FL 32746, United States; Ontario Institute for Cancer Research, Toronto, ON M5G 0A3, Canada; Division of Medical Oncology, BC Cancer, Vancouver, BC V5Z 4E6, Canada; Sarcoma Cancer Foundation of Canada, Toronto, ON M4V 1L5, Canada; Drug Development Program (Phase I), Princess Margaret Cancer Centre, University Health Network,Toronto, ON M5G 2M9, Canada; Oncology Clinical Trials, Research Institute of McGill University Health Centre, Montreal, QC H4A 3J1, Canada; Drug Development Program (Phase I), Princess Margaret Cancer Centre, University Health Network,Toronto, ON M5G 2M9, Canada; Clinical Development Operations Canada, Boehringer Ingelheim Canada Ltd, Burlington, ON L7L 5H4, Canada; Clinical Development Operations Canada, Boehringer Ingelheim Canada Ltd, Burlington, ON L7L 5H4, Canada; Clinical Development Operations Canada, Boehringer Ingelheim Canada Ltd, Burlington, ON L7L 5H4, Canada; Drug Development Program (Phase I), Princess Margaret Cancer Centre, University Health Network,Toronto, ON M5G 2M9, Canada; Drug Development Program (Phase I), Princess Margaret Cancer Centre, University Health Network,Toronto, ON M5G 2M9, Canada

**Keywords:** phase 1 oncology trials, Canada, global clinical trial leadership, participant-centric trial design, CANLead

## Abstract

**Background:**

Phase 1 oncology clinical trials have become increasingly complex, resource-intensive, but essential to modernize drug development. Despite strong academic infrastructure, Canada remains underrepresented in early-phase cancer trials relative to international peers.

**Methods:**

To advance this vision, this perspective summarizes barriers and opportunities impacting the conduct of phase 1 oncology clinical trials in Canada, based on discussions from sponsor, patient advocate, ethics, and clinical research site representatives across national major phase 1 units at the inaugural “Practical Workshop of Conducting Phase 1 Trials in Canada.”

**Results:**

Current challenges include overly restrictive and complex trial design that increases participant burden, study activation delays, operational inefficiencies, and limited access to investigational therapies following disease progression. However, Canada’s integrated academic cancer centers, publicly funded healthcare system, and ongoing advances in harmonization strategies present opportunities to strengthen national competitiveness in global site selection.

**Conclusion:**

Participant-centric strategies, earlier collaboration amongst stakeholders, and targeted operational improvements at local, provincial, and national levels are crucial to elevate **Can**ada as a global **lead**er (CAN*L*ead) in phase 1 oncology clinical research. Together, the approaches position Canada to advance its competitiveness and take a more prominent role on the international stage, while also providing a practical framework for developing and optimizing phase 1 units globally.

Implication for Practice The actionable strategies outlined provide a multi-faceted, participant-centric model for phase 1 oncology trial design and management. Informed by sponsor, site, patient advocate, ethics board, and other trial stakeholders, these insights emerged from Canada’s first collective discussion of successful phase 1 trial conduct. The framework broadens patient access, accelerates trial activation, enhances participant-focused design, and improves operational efficiency, while upholding high-quality care. It provides a scalable blueprint for counties developing phase 1 programs, informs future trial design, and showcases how Canada is advancing its global competitiveness and leadership in early-phase cancer clinical trials.

## Introduction

Canada is recognized internationally for excellence in clinical research, ranking third globally in the number of new clinical trials initiated, leading the G7 in studies per capita.^1^ Its established clinical expertise, public research infrastructure, and active participation in international advisory boards uniquely position Canada to make meaningful contributions, yet it remains underrepresented in phase 1 oncology clinical trials. This does not reflect limited scientific capability, rather structural, operational, and system-level barriers.

**Figure 1.**
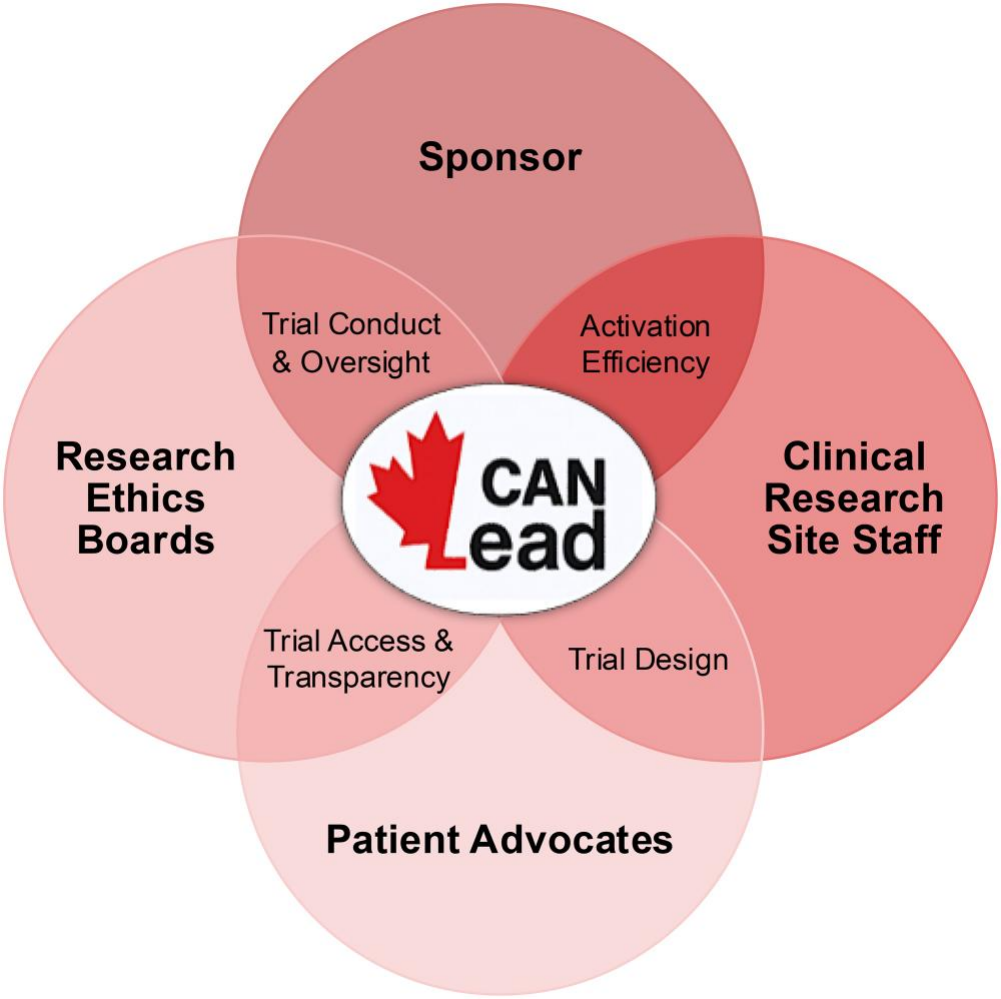
Overview of CAN*L*ead network. This diagram highlights the key focus areas for optimization within phase 1 oncology clinical trials and illustrate how each stakeholder’s specialized expertise strengthens CAN*L*ead’s emerging collaborative network.

Our current work draws from sponsor, site staff, patient advocate, research ethics board (REB) representatives across 7 major Canadian phase 1 clinical trial/drug development units through the inaugural “Practical Workshop of Conducting Phase 1 Trials in Canada,” held on April 4, 2025. This constitutes the first coordinated nation-wide effort focused on phase 1 cancer clinical trials in Canada and the establishment of our emerging network (CAN*L*ead; Figure 1). Through this multidisciplinary knowledge exchange, we identified key elements defining high-performing phase 1 units, constraints to early-phase cancer trials in Canada, and opportunities to better position **Can**ada as a global **lead**er (CAN*L*ead) in phase 1 oncology research.

## System-level barriers to phase 1 oncology trials in Canada

Phase 1 oncology clinical trials are critical for translating novel anticancer agents from preclinical development to first-in-human testing, establishing recommended doses and schedules, and evaluating toxicities. Increasingly, they have restrictive eligibility criteria, intensive biospecimen sampling, invasive procedural requirements (eg, multiple mandatory biopsies when archival tissue may suffice), frequent participant-reported outcome assessments, and diagnostic testing with limited scientific justification, collectively driving trial complexity.^2^

Coupled with Canada’s 11.8-year median timeline of drug development to market, sponsors often combine multiple scientific questions into one protocol and prioritize activation speed, recruitment metrics, and data quality when selecting countries for trial participation.^3^ In Canada, delays related to ethics review, contract negotiations, and site readiness (eg, redundant qualification requirements, frequent protocol amendments, prolonged internal reviews) can significantly shorten recruitment windows and impair global competitiveness. While Health Canada and Canadian REBs generally review trials within 30 days, this period does not always culminate in full approval. Teo et al.^4^ reported a 17-week average activation timeline across Canadian trial sites. In contrast, faster international approval benchmarks (eg, 2 weeks in Australia, 3 weeks in South Korea) impact sponsors’ site selection and first-participant enrollment.^5^^,^^6^

High-quality trial conduct depends on well-trained, proactive site, sponsor, and Contract Research Organization (CRO) teams, which can be compounded by the complexity of phase 1 studies. Frequent CRO turnover and limited oncology-specific expertise contribute to poor knowledge transfer, workflow redundancies, and increased burden on sites and sponsors, undermining trial efficiency.^7^

For patients, Canadian enrollment is also challenged by structural inequities, including disparities in geographic distribution of research hospitals and, until recently, limited national ethics harmonization, compared to European countries.^8–10^ As reported by Kasner et al.,^11^ lower enrollment per trial often necessitates activating additional studies to meet accrual targets.

Enrollment is further hindered by limited public awareness of phase 1 clinical trials and misinformation—often amplified by social media—diminishing trust and scientific literacy.^12^ Intersectional barriers, geographic distance from treatment centers, financial and childcare constraints, along with trial waitlists and competitive slot allocation exacerbate these inequities, particularly for patients with rapidly progressive disease.^13^ Collectively, this pressures sites to enroll suboptimal participants to avoid forfeiting allocated slots.

Lastly, end-of-treatment is a particularly vulnerable period for participants, often marked by disease progression. Early trial termination due to lack of efficacy, transfer of trial sponsorship, or discontinuation of drug access programs poses ethical and emotional challenges, especially where clinical benefits have been observed. Participants often feel disheartened by the limited communication surrounding study closure and the burden of extensive follow-up assessments.^14^ Logistically, delayed payments and closure timelines—largely from extended safety follow-ups–further strain site resources and unnecessarily prolong trial termination.

## Strengthening phase 1 trials, units, and national impact

Canada is uniquely positioned to expand its global leadership in phase 1 oncology trials, leveraging centralized ethics models, integrated academic centers, and emerging referral networks, supported by the publicly funded healthcare system. The following strategies provide a multi-level actionable framework to enhance trial design, operational efficiency, and participant-centric care (see Table 1 for specific examples).

**Table 1:** Summary of CAN*L*ead recommendations for successful phase 1 clinical trial and unit development.

**1.0**	**Practical considerations for protocol development**	
	1.1	***Early pipeline engagement for effective protocol development*** Expand early pipeline discussions, particularly during protocol synopsis, to include investigators, beyond those traditionally selected by sponsors (eg, Partners of Choice), site staff, and patient advocates.
	1.2	***Modernize trial designs with participant-centric approaches*** Proposed strategies that balance participant burden with scientific rigor through flexible eligibility criteria and procedural schedules: Customized laboratory tests aligned with study drug mechanism of action Clarification of mandatory vs optional biopsies Equitable inclusion across demographic variables (eg, race, ethnicity, age, sex, gender) Broader eligibility for prior lines of therapy and toxicity allowances, when feasible, and not impactful on study drug assessment Expanded inclusion of tumor types with the same mutation specified in the protocol and tumor-agnostic cohorts, once predefined enrollment targets are met to increase trial opportunities for participants with rare cancers Provision of participant support services (eg, trial visit reimbursement for travel/parking and meals, concierge for travel) promotes equitable trial participation Careful assessment of currently extensive end-of-treatment requirements and post-trial safety monitoring, being mindful of participants’ physical, mental, and emotional burden during trial discontinuation^15^ Reduced reliance on waitlists and competitive slot allocations, where applicable
	1.3	***Integrate compassionate care access and crossover opportunities*** Extend study drug provisions for participants demonstrating clinical benefit and outline compassionate access upfront, especially in cases of disease progression (eg, combination therapy crossover when monotherapy proves ineffective), cohort closure, or trial termination.
	1.4	***Ensure transparent and timely closeout*** Maintain timely closeout procedures, including data cleanup and site payments. Share rationale for termination and key safety findings with sites and participants.
**2.0**	**Operational best practices**	
	2.1	***Enhance site activation readiness through regulatory agility*** Early operational alignment between sites, sponsors, ethics board, and CROs can streamline start-up processes, reduce bottlenecks, and enhance global competitiveness through: Parallel section-by-section reviews for contracts and budgets Early disclosure of site feasibility and resource needs Internal meetings between investigator and ethics board coordinator(s) prior to regulatory submissions for proactive resolution of potential issues Early provision of a complete start-up package (ie, finalized protocol, investigator’s brochure, all manuals, and all participant-facing materials), prior to the initial regulatory submission to significantly expedite the ethics board approval to site activation timeline
	2.2	***Important of multi-stakeholder communication*** Foster open communication and transparency amongst trial participants, sites, sponsors, and CROs, including, but not limited to, accurate operational and trial requirements, regulatory and study management, and safety concerns. Actionable approaches for stakeholders include: ***Sponsors**:* Facilitate regular global teleconferences to review of adverse events and biospecimen analyses to encourage collaborative discussions on dose escalations, optimizations, and modifications, as well as toxicities and prophylactic management Provide advanced notice on slot assignment with reasonable screening window (eg, 2 weeks) for sites to adequately assess participant eligibility Provide sites with sufficient time for data locks sites and with frequent, up-to-date regulatory, data entry, and query resolution metrics, accounting for non-conformant data and system errors Minimize unnecessary trainings for site personnel (eg, sponsor-specific portals that reiterate protocol information, completing sponsor-mandated training for non-local adverse event acknowledgement that investigators will not directly use) and signing of ancillary items (eg, receipt acknowledgements)^7^ When a CRO is used, prioritize selection due to the frequent staff turnover, facilitate specialized phase 1 oncology and protocol-focused trainings, emphasize high-quality communication, and define their scope to minimize inefficiencies^7^ ***CROs**:* Minimize study monitor turnover, where possible Provide sites with greater transparency on sponsor-CRO processes (eg, workflow, turnaround timeline, sponsor expectations) Ensure timely communication between site and sponsor, particularly for regulatory submissions, prompting sponsor as appropriate to avoid operational delays Conduct a preliminary review of queries generated by the sponsor and data management teams to minimize previously addressed issues and confirm whether additional clarification is warranted from sponsor end, before sending them to site personnel Facilitate effective knowledge transfer during study monitor changeover to reduce repetitive requests asked to site personnel Conduct site-study monitor visits when appropriate per participant safety and key data milestones, rather than for CRO-mandated guidelines^7^ Avoid overclassification of minor deviations as major deviations; stratify based on their impact on participant safety and data integrity^7^ ***Sites**:* Establish strong relationships with academic colleagues—local and in community centers—and participant groups Allocate sufficient time to discuss extensive study demands, longevity, and quality-of-life expectations with potential phase 1 trial participants/families to improve digital literacy Educate research teams on emerging studies and associated toxicities, trial slot allocation models, and common participant barriers Liaise with ethics board and patient advocacy groups to design targeted educational resources to strengthen public trust and literacy in the ethics review process of early-phase studies Advocate against unsafe dose escalations to prevent undue toxicities for current and future study participants, despite sponsor pressure to advance the trial Maintain good documentation practices with timely reporting of adverse events and dose-limited toxicities
	2.3	***Optimize study conduct with specialized operational models and dedicated clinical roles*** Differential operational models of phase 1 units can address challenges in study conduct — each adaptable based on their referral networks, staffing, resources, and site capacities: *Mothership model*: Centralized lead investigator manages participants—agnostic of cancer type, which maximizes expertise but relies on strong referral networks from other disease groups and community oncologists for escalation cohorts *Hub and Spoke model*: Disease site groups (eg, breast, lung, etc.) manage recruitment and study conduct, while the phase 1 unit serves as an operational hub, which increases buy-in, especially for expansion cohorts, but can dilute lead investigator oversight *Hybrid model*: Dose escalation utilizes a *Mothership model* and transitions to a *Hub and Spoke model* for expansion cohorts, but challenges may include increased regulatory burden when changing lead investigator mid-study Specialized roles within these models bridge the gap between clinical research and participant well-being, while optimizing efficiencies. They which include: Dedicated phase 1 pharmacist to operationalize complex treatment regimens and standardize practice across earl-phase oncology trials Clinical trials nurse specialist to guide newly referred patients through trial processes, enhance understanding of research participation, and coordinate essential support with the healthcare team
**3.0**	**Actionable local, provincial/regional, national capacity-building strategies**	
	3.1	**Local approaches**
	3.1.1	***Strengthen site leadership, phase 1 training, and unit capacity*** Prioritize targeted investment in specialized infrastructure (eg, nuclear medicine and lab facilities), overnight monitoring (eg, for potential cytokine release syndrome), weekend biospecimen sampling, robust research operating procedures, referral networks, and dedicated support (eg, investigational pharmacy, correlative science, biospecimen, and radiology) to streamline activation and sustain long-term phase 1 trial pipeline engagement. Implement specialized phase 1 training and mentorship opportunities for early-career investigators and research staff to ensure high-quality trial execution, elevate site credibility, and build sponsor trust, with emphasis on: Identification on suitable trial patients Effective consent practices, educating participants without overwhelming them Soft skill and leadership development (eg, North America Star Consortium’s ARTES Program: Le**A**dership Development Cou**R**se for Early Career Investiga**T**ors with Interactive Group L**E**arning and Individualized Coaching **S**ession, established in 2021) Building expertise on trial and cohort selection based on available patient population and resources (eg, expansion cohorts offering enrollment, operational and financial stability as dose is optimized) Engagement in ethics board activities to build regulatory knowledge
	3.1.2	***Utilize site-centered trial management systems and operational tools to streamline study conduct*** Pre-site initiation visit protocol reviews improve start-up efficiency and staff competency, supporting trainee education as early-career investigators present key protocol elements to the multidisciplinary study team Weekly phase 1 program meetings, daily nursing huddles, and early-career investigator-led handover emails strengthen trial oversight and ensure timely updates on participant status, especially helpful for those newly enrolled or experiencing toxicities Site-centered clinical trial management systems, such as Phase One Spot Tracker (POST), enables real-time tracking of participant status, slot availability, and regulatory submissions—implemented at select Canadian sites, supporting coordinated trial oversight^18^ Platforms that integrate user-friendly, patient referral functionality (eg, Canadian Cancer Clinical Trials Network (3ctn.ca); in development for Phase One Spot Tracker)
	3.2	**Provincial/regional and national approaches to compete globally**
	3.2.1	***Create, adopt, and scale effective multi-level harmonization strategies*** Develop and implement strategies to expedite regulatory review, institutional approval, and site readiness across site, provincial, and national levels: *Centralized ethics model*: Provincial ethics boards (eg, Ontario’s cancer-specific REB (ocreb.ca), Quebec’s CATALIS (catalisquebec.com)—FAST TRACK Evaluation Service and national initiatives (eg, Accelerating Clinical Trials’s pan-Canadian REB initiative: CanReview, (canreview.ca)) streamline multi-site trial reviews^19^^,^^20^ *Expedited departmental review initiatives*: Accelerated site approval pathways decrease activation timelines improve regulatory and operational agility, as successfully piloted at Princess Margaret Cancer Centre with 6-week initial application approval timelines (results underway) *Standardized clinical trial documents*: Leveraging standardized consent form templates, as done at Ontario’s cancer-specific ethics board (ocreb.ca), and trial agreements relating to site participation and data sharing/sample transfers through ACT (act-aec.ca) reduces administrative burden across stakeholders, only needing to modify trial-specific information

### Protocol development and participant-centric trial design

Early engagement of investigators, site staff, and patient advocates during protocol development optimizes trial design, allowing refinement of eligibility criteria, safety assessments, and crossover opportunities. It facilitates proactive resource planning and minimizes trial conflicts. Aligned with Project Optimus—recent dose optimization initiative in oncology drug development—these promote collaborative discussions on dose identification and modification to maximize clinical and non-clinical data, reduce protocol amendments, and support compassionate care.^15^

Participant-centric trial designs balance scientific rigor with trial burden, as reinforced by the *Methods in Clinical Cancer Research Workshop* model, where investigators justify study procedures and visit schedules to patient advocates.^16^ Investigators can reference our supplemental resource to help assess effective study designs and strengthen their phase 1 trial competency (Table S1).

### Operational best practices

Regulatory and site activation timelines are critical determinants of global site selection and may indicate trial success, requiring operational alignment among all stakeholders.^17^ Early disclosure of site capabilities and resource needs facilitates accurate feasibility assessments and mitigates downstream bottlenecks. Continued alignment during parallel sponsor-site reviews in contract and budget negotiations helps maintain momentum and support strategic budgeting to attract global investment in Canada.

The most effective approach to timely site activation is the sponsor’s early provision of the complete start-up package prior to initial regulatory submissions. This includes the finalized protocol, investigator’s brochure, manuals (eg, pharmacy, lab, imaging), and participant-facing materials (eg, informed consent forms, medical diaries, questionnaires), with emphasis on the finalized pharmacy manual, for which sponsor-site collaboration on its development is welcomed. Sponsors should also provide advanced notice and adequate screening timelines for slot assignments to support an equitable enrollment system and sustainable trial management.

Specialized phase 1 staff are key to maintaining the trial momentum. A dedicated phase 1 pharmacist has the expertise to assess drug interactions and determine the feasibility of study drug preparation and proposed administration conditions, serving as a great resource to support other oncology trial pharmacists nationwide. A clinical trials nurse specialist can similarly streamline trial efficiencies and enhance participant experience by developing referral networks, supporting participants’ trial knowledge, and coordinating essential support with social workers and other healthcare team members, helping them navigate the emotional and physical demands of trial participation. Leveraging technology through participant-focused and trial management tools enhances transparency and trial oversight. For a thorough guide on early-phase oncology trial roles and scope, refer to the *Society of Clinical Research Sites’ Global Oncology Program* (myscrs.org/wp-content/uploads/2023/01/Phase-1-Draft-Document_V71.pdf).

### Local, provincial, and national capacity-building strategies

At an institutional level, development or investment in site-centered clinical trial management systems and expedited department review initiatives can promote sustainable trial oversight of regulatory timelines and participant status, while substantially reducing activation timelines.^18^ Strong site leadership, phase 1-specific training, mentorship, structured program communications, standardized consent form templates, and targeted investment in specialized infrastructure prove to be crucial for high-quality operational readiness.

Provincial REBs, like Ontario’s centralized cancer ethics board (ocreb.ca), streamlines multicenter clinical trial reviews, while Quebec’s CATALIS FAST TRACK Evaluation Service (catalisquebec.com) involves intensive sponsor-site collaboration to achieve expedited regulatory approvals within 8 weeks or less at public institutions.^19^

Nationally, Accelerating Clinical Trials (ACT; act-aec.ca) is a federally funded initiative that launched CanReview (canreview.ca) in May 2025 as a pan-Canadian distributed REB-of-Record model that enables a single ethical submission for multicenter randomized controlled trials. Coupled with ACT’s standardized agreement for site participation and data sharing/sample transfer, these simplified regulatory and activation processes nationwide—the latter of which already demonstrating efficiencies with contract activations in as little as 10 days.^20^ Alongside the 50+ institutions adopting ACT’s initiatives and emerging sponsor-led master clinical trial agreements, these efforts reflect meaningful progress toward a more unified and globally competitive Canadian clinical trial landscape.

Further initiatives include sponsor-funded trainee programs, such as Investigational New Drug fellowships for resource-limited sites, scientific writing projects with equitable authorship, and specialized phase 1 trial training programs to further strengthen Canada’s early-phase oncology research workforce.

## Conclusion

CAN*L*ead is the first national phase 1-specific oncology network, complementing initiatives like Canadian Cancer Clinical Trials Network (3ctn.ca) and UK’s Experimental Cancer Medicine Centre Network (ecmcnetwork.org.uk). With early stakeholder engagement, operational alignment, and improved communication, CAN*L*ead aims to expand trial capacity, enhance participant experience, and solidify Canada’s leadership in early-phase oncology research, strengthening site-sponsor partnerships for long-term sustainability.

With overwhelmingly positive feedback on the inaugural CAN*L*ead workshop (93% response rate, >90% satisfaction, 97% supportive of annual continuation), future workshops will be adaptable. The March 2026 event focused on national bottleneck mapping, referral network harmonization, financial sustainability, and global trial opportunities.

Our actionable strategies provide a multi-faceted, participant-centric model for phase 1 trial design and management. Developed through Canada’s first collective discussion with sponsor, site, patient advocate, and REB personnel, these approaches broaden drug access, accelerate trial activation, and improve operational efficiency. They offer a practical framework for countries developing phase 1 programs, while demonstrating Canada’s enthusiasm and efforts toward advocating for its global position in early-phase cancer research.

## Supplementary Material

oyag190_Supplementary_Data

## Data Availability

All relevant data are contained within the publication itself.
