# Supplementary material for: CANLead: Canada as a Phase 1 Clinical Trial Leader — Opportunities, Initiatives, and Collaborative Innovation to Elevate National Competitiveness
Source: Oncologist. 2026 May 22;31(7):oyag190. doi: 10.1093/oncolo/oyag190 (PMC13274539; doi:10.1093/oncolo/oyag190)
Supplement: oyag190_Supplementary_Data [file oyag190_supplementary_data.docx]

**Supplemental Information**

**CAN*L*ead: Canada as a Phase 1 Clinical Trial Leader – Opportunities, Initiatives, and Collaborative Innovation to Elevate National Competitiveness**

Ashley A. Adile, Gagan Sran, Philippe Bedard, Lindsay Carlsson, Jeffrey Doi, Nancy Drummond-Ivars, Sevan Hakgor, Amy Henderson, John F. Hilton, P. David Josephy, Jim Kremidas, Jacqueline Limoges, Daniel J. Renouf, Fiona Ross, Abdulazeez Salawu, Ramy Saleh, Lillian L. Siu, Isabelle Voccia, Hanusya G. Lewis, Miney Paquette, Albiruni R. Abdul Razak, and Anna Spreafico

**Supplemental Table S1. Examples of Considerations to Evaluate Phase 1 Clinical Trial Design**

| Is the trial ethically sound? | Is the trial scientifically valid? | Is the trial practical to conduct? |
| --- | --- | --- |
| Does the study preclude participants from access to approved agents/standard of care/best supportive care in Canada? | Are the study objectives, design and methods scientifically sound and appropriate to address the research questions? | Does the site have all the required resources to conduct the trial? |
| *Example: Anti-W inhibitors cannot be used prior to study A, investigating melanoma participants; however, inhibitor W is standard treatment for advanced melanoma. Will the participant lose access to the best supportive care?* | *Example: All non-clinical data have tested the* *study drug in lung models; however, the first-in-human study is selective for renal cell carcinoma only. Can one extrapolate such non-clinical data across species?* | *Example: Evening biospecimen sample collection is required for study B, but does the site have the appropriate research staff?*  *Example: Study drug in study has a high risk of cytokine release syndrome, but does the site have access to an intensive care unit for monitoring?* |
| Are all required procedures ethical? ​ | Does the study design take into consideration factors linked to the compound/drug? | ​What are the potential logistical challenges? Can these barriers be overcome? |
| *Example: Fresh tumour biopsy is mandatory for prescreening. If the tumor is negative for biomarker X, the patient cannot be enrolled in the study but went through an unnecessary invasive procedure.* | *Example: Based on the number of biospecimen collection timepoints, dose levels to investigate, participants to be dosed, and non-evaluable participants, is there any flexibility to change the study design, as clinical data are generated, to be more agile?* | *​Example: Specific genetic testing is not standard of care for all participants in some tumour types. Does the sponsor provide support for central testing?* |
| Do some required procedures pose a higher known risk of harm to participants? If so, what mitigation strategies are in place in the protocol? | Are there biases in the protocol, and is the study designed to minimize them? | Can all required procedures be implemented at the site? ​Is there any room for improvement in the eligibility criteria? |
| *Example: Antibody Y is expected to increase risk of cytokine release syndrome. Are pre-medications considered? When? Is the protocol language flexible to allow priming dose implementation?* | *Example: Confirm if there is selection bias, such as where the study drug is intended to reverse resistance to inhibitor Z, but the study is enrolling for participants who have not received inhibitor Z previously, which contradicts the proposed mechanism of action.* | *Example: Study drug is delivered via intra-tumoral injection – both superficial and visceral. Does the site have* *the expertise and experience? Is interventional radiology expertise on site?* |
